# Atopic children and use of prescribed medication: A comprehensive study in general practice

**DOI:** 10.1371/journal.pone.0182664

**Published:** 2017-08-24

**Authors:** David H. J. Pols, Mark M. J. Nielen, Arthur M. Bohnen, Joke C. Korevaar, Patrick J. E. Bindels

**Affiliations:** 1 Department of General Practice, Erasmus MC, University Medical Center Rotterdam, Rotterdam, The Netherlands; 2 NIVEL, Netherlands Institute for Health Services Research, Utrecht, The Netherlands; University of Southampton School of Medicine, UNITED KINGDOM

## Abstract

**Purpose:**

A comprehensive and representative nationwide general practice database was explored to study associations between atopic disorders and prescribed medication in children.

**Method:**

All children aged 0–18 years listed in the NIVEL Primary Care Database in 2014 were selected. Atopic children with atopic eczema, asthma and allergic rhinitis (AR) were matched with controls (not diagnosed with any of these disorders) within the same general practice on age and gender. Logistic regression analyses were performed to study the differences in prescribed medication between both groups by calculating odds ratios (OR); 93 different medication groups were studied.

**Results:**

A total of 45,964 children with at least one atopic disorder were identified and matched with controls. Disorder-specific prescriptions seem to reflect evidence-based medicine guidelines for atopic eczema, asthma and AR. However, these disorder-specific prescriptions were also prescribed for children who were not registered as having that specific disorder. For eczema-related medication, about 3.7–8.4% of the children with non-eczematous atopic morbidity received these prescriptions, compared to 1.4–3.5% of the non-atopic children. The same pattern was observed for anti-asthmatics (having non-asthmatic atopic morbidity: 0.8–6.2% vs. controls: 0.3–2.1%) and AR-related medication (having non-AR atopic morbidity: 4.7–12.5% vs. controls: 2.8–3.1%). Also, non-atopic related medication, such as laxatives and antibiotics were more frequently prescribed for atopic children.

**Conclusions:**

The present study shows that atopic children received more prescriptions, compared to non-atopic children. Non-atopic controls frequently received specific prescriptions for atopic disorders. This indicates that children with atopic disorders need better monitoring by their GP.

## Introduction

Many children are diagnosed with atopic disorders [1, 2] and are likely to consult their general practitioners (GP) for atopic-related symptoms. In the present study, we refer to atopy as one or more of the following established diagnoses: atopic eczema, asthma and/or allergic rhinitis (AR).

Evidence-based medicine guidelines support Dutch GPs in the decision-making process when prescribing medication [3–5]. According to these guidelines, the cornerstone for the treatment of atopic eczema in children are emollients and corticosteroid crèmes, prescribed in a stepwise approach [3]. When anti-asthmatic inhalation medication is needed, a GP will start with a short-acting beta agonist, followed by inhaled corticosteroids when indicated [4]. For AR, treatment will depend on the severity of symptoms. Intermittent symptoms are often treated with local or oral antihistamines on demand, while moderate to severe symptoms will be treated with corticosteroid nasal sprays [5]. How often these atopic-related prescriptions are also given to children that are not labeled/diagnosed with a specific atopic disorder is not yet known and could reflect underdiagnosis or insufficient coding.

Atopic disorders are associated with comorbidity [6], and this can result in non-atopic related prescriptions for these atopic children as well. However, to what extent these atopic children have a higher risk to receive more (non-) atopic related prescriptions has not yet been examined in primary care. Knowing more about these differences can help a GP to provide better care for his atopic patients.

Therefore, in this study, an extensive and representative nationwide general practice database was used to investigate associations between atopic disorders and prescribed medications. Two research questions were formulated: i) Which medications are prescribed by GPs for atopic disorders? ii) What kind of other medications do atopic children receive?

## Methods

### Study population

All non-institutionalized Dutch inhabitants are compulsorily listed with a general practice, including patients who do not visit their GP on a regular basis. The Netherlands Institute for Health Services Research-Primary Care Database (NIVEL-PCD) uses the electronical health records (EHRs) of all listed patients in participating practices for research purposes. The data are representative for the Dutch population [7] and based on routinely recorded data (type of consultation, morbidity, and prescriptions). In 2014, about 500 general practices participated, including data of about 1,700,000 patients (www.nivel.nl/en/dossier/nivel-primary-care-database), i.e. over 10% of the total Dutch population. Morbidity is recorded by GPs (frontline for the Dutch healthcare system) using the International Classification of Primary Care-1 (ICPC-1). This is a classification method for primary care and accepted by the WHO [8]. Relevant consultations, prescriptions and referrals are clustered in ICPC classified episodes of care. Atopic episodes of care are labeled with ICPC codes: S87 (atopic eczema), R96 (asthma) and R97 (allergic rhinitis). ICPC codes specific for food allergies are not available.

Only data from EHRs of general practices with sufficient data quality were used. They had to fulfill the following criteria: at least 500 listed patients (standard practice size: 2350 patients), complete morbidity registration (defined as ≥ 46 weeks per year) and sufficient ICPC coding (defined as ≥ 70% of the recorded disease episodes labeled with an ICPC code). To reduce the risk of registration bias for atopic disorders, a minimum follow-up of 3 successive years was required for each child (age range 0–18 years), including 2014. We considered a 3-year follow-up sufficient time for a GP to diagnose a child with atopic disorders, since a Dutch GP sees about 72% of pediatric patients at least once a year [9]. The following descriptive data were routinely collected: gender, year and quarter of birth, period in which the individual child was registered in the general practice, and the unique code of the general practice.

### Identification of atopic children

When available, the EHRs from 2002–2014 were examined in order not to miss any relevant atopic diagnosis. Because there is a risk of misclassification (GPs work with probability diagnoses), ICPC codes and their related episodes of care were corrected in order to select cases with a higher probability of a clinically relevant disorder. In practice, an atopic episode of care was maintained if (based on available data from EHRs in the period 2002–2014) the child had at least two contact moments in that episode of care and had received at least two relevant prescriptions. If the child did not meet these criteria, the child was considered not to have that atopic disorder [ref NIVEL 1]. It was not a requirement that the patient had visited the GP in 2014 for that specific atopic disorder.

### Atopic triad

A forth distinct group of children, with all three atopic disorders, might exist according to a meta-analysis [1]. This is in contrast to the traditional classification of children with atopic eczema or asthma or AR. ‘Atopic triad’ episodes were developed for research purposes in order to learn more about this potentially unique group of children and were only created when a child was diagnosed with all three atopic disorders(based on available data from EHRs in the period 2002–2014).

### Studied medication

Primary care physicians recorded prescriptions using the Anatomical Therapeutic Chemical (ATC) Classification System. This system is controlled by the World Health Organization Collaborating Centre for Drug Statistics Methodology (WHOCC), and was first published in 1976. All ATC codes were examined at the second level, indicating the therapeutic main group and consisting of two digits. In some cases, a subgroup analysis was done at the ATC 3 level, indicating the therapeutic/pharmacological subgroup and consisting of one letter. All 93 ATC codes at the ATC 2 level were studied (S1 Table). Prescription data from 2014 were examined.

### Design

In a nested case-control study design, for each atopic child one matched control patient was selected (not diagnosed with an atopic disorder) within the same general practice, based on age and gender (in 2014). In order to include as many pairs of cases and controls as possible, a 1:1 ratio was chosen. This allows the results to carry more weight and make the conclusions better generalizable to future populations. When using a 1:2 ratio, over 40% of the cases had to be dropped.

### Statistical analyses

To study associations between the presence of atopic disorders and prescriptions in children, logistic regression analyses were performed for children that solely had atopic eczema, asthma, or AR and therefore no other atopic comorbidity. The same analyses were performed for the atopic triad. As a result of multiple testing, the level of significance was set on p≤0.001. Modifying effects of age and gender were tested for all associations. When the effect was significant (p≤0.01), associations were also presented for subgroups for age (0–6 vs. 7–12 vs. 13–18 years) and gender (boy vs. girl). All analyses were conducted in Stata 13 and Excel 2010. Prevalences are presented in percentages.

### Ethical approval

Dutch law allows the use of EHRs for research purposes under certain conditions. According to this legislation, it is not necessary to obtain informed consent from patients or approval from a medical ethics committee for this type of observational study that contains no directly identifiable data (Dutch Civil Law, Article 7: 458). No waiver of ethical approval was therefore obtained by an Institutional Review Board or ethics committee. The authors did not have access to identifying information at any moment during the analysis of the data.

## Results

### General characteristics

In 2014, 409,312 children were identified for this study, including 45,964 atopic children with at least one atopic disorder (selection of cases with higher probability of a clinically relevant disorder) and 3-year follow-up; these children were from 316 different general practices participating in NIVEL-PCD, for which 45,964 non-atopic children were matched (Table 1). The mean age was 10.0 (SD 4.9) years. Of all atopic children, 52.7% were male. For solely atopic eczema, asthma, AR and the atopic triad, 48.2%, 59.0%, 57.8% and 61.4% were male, respectively.

**Table 1:** General characteristics.

	Index patients (n = 45,964)	Control patients (n = 45,964)
Males	52.7%	52.7%
Mean age (SD), years	10.0 (4.9)	10.0 (4.9)
Age 0–6 years	29.5%	29.5%
Age 7–12 years	35.2%	35.2%
Age 13–18 years	35.3%	35.3%
Atopic eczema	21,285	0
Only atopic eczema*	15,530	0
Asthma	13,196	0
Only asthma*	7,887	0
Allergic rhinitis	11,483	0
Only allergic rhinitis*	6,835	0
Atopic triad	559	0

* the child did not have any of the other atopic disorder

### Children registered with only atopic eczema

A child with atopic eczema received on average 1.5 different prescriptions in 2014, compared to 0.7 different prescriptions for the controls; this difference was significant (Table 2). In total, 61% of all children with atopic eczema did not receive relevant medication for atopic eczema in 2014. The highest ORs (12.5–13.1) were observed for atopic eczema related medication: emollients (D02) and dermatological corticosteroids (D07) (Table 3).

**Table 2:** Number of different prescriptions received in 2014.

Disorder	Index patients	Control patients
Only atopic eczema*	1.5	0.7
Only asthma*	1.8	0.7
Only allergic rhinitis*	2.2	0.8

* the child did not have any of the other atopic disorder

**Table 3:** Significantly (p≤0.001) associated medication in children registered with only atopic eczema (Ec) versus controls (non-atopic children) (n = 31,060).

ICPC	OR	95%-CI	Prevalence (%)		OR per sex group		OR within age			Description ICPC codes
			Ec	No atopy	boy	girl	0–6	7–12	13–18	
**Atopic eczema related medication**										
D02	13.09	11.58–14.80	19.85	1.87			11.68	12.63	20.00	Emollients and protectives ^†^
D07	12.52	11.45–13.68	32.91	3.78			11.18	11.87	16.80	Corticosteroids, dermatological preparations ^†^
**Asthma related medication**										
R03	1.97	1.72–2.26	3.98	2.07						Anti-asthmatics
**Allergic rhinitis related medication**										
R01	1.54	1.37–1.73	4.72	3.12						Nasal preparations
R06	2.90	2.59–3.24	7.72	2.81						Antihistamines for systemic use
**Medication related to atopic disorders**										
A06	1.35	1.21–1.50	5.48	4.13						Laxatives
J01	1.35	1.26–1.44	15.30	11.86						Antibacterial for systemic use
N05	1.43	1.17–1.75	1.48	1.04						Psycholeptics
S02	1.48	1.30–1.68	3.83	2.63						Otologicals
S01	1.64	1.46–1.84	5.14	3.21						Ophthalmologicals
D01	1.68	1.50–1.87	5.85	3.57						Antifungals for dermatological use
D06	1.87	1.71–2.05	9.29	5.21						Antibiotics and chemotherapeutics for dermatological use
D04	1.89	1.34–2.65	0.62	0.33						Antipruritic, including antihistamines, anaesthetics, etc.
L04	***2*.*37***	***1*.*17–4*.*80***	***0*.*17***	***0*.*07***			0.50	1.00	6.36	Immunosuppressive agents ^†^
D08	2.64	1.69–4.11	0.46	0.17						Antiseptics and disinfectants
D11	2.79	2.24–3.47	1.94	0.71						Other dermatological preparations
D05	4.11	2.06–8.21	0.26	0.06						Antipsoriatics
C01	6.44	3.73–11.10	0.62	0.10						Cardiac therapy (e.g. epinephrine auto-injectors)

^†^ significant (p≤0.01) influence of age

**Italic**: overall model not significant

Other dermatological preparations were also frequently prescribed, e.g. antifungals (OR 1.7), antipruritics (OR 1.9), antipsoriatics (OR 4.1), antibiotics (OR 1.9), antiseptics (OR 2.6) and other dermatological preparations (OR 2.8), e.g. agents for dermatitis, excluding corticosteroids. Although less frequently prescribed, a high OR of 6.4 was observed for ATC code C01 (88% concerned epinephrine auto-injectors)(Table 3).

Children with atopic eczema received significantly more emollients (OR 11.7->20.0) and dermatological corticosteroids (OR 11.2->16.8) at older age. This also applied for immunosuppressive agents (OR 0.5-> 6.4). Sex did not influence prescriptions in children with atopic eczema.

Eczema-related medication was also prescribed for children that were not registered as having atopic eczema. For eczema-related medication, about 3.7–8.4% of the children with atopic comorbidity received these prescriptions compared to 1.4–3.5% of the non-atopic children. Anti-asthmatics were used by 4% of the children with atopic eczema (OR 2.0) even though the GP did not register them as having asthma. This same pattern is seen for medication related to AR (OR 1.5–2.9) (Table 3).

### Children registered with only asthma

A child with asthma received on average 1.8 different prescriptions in 2014 compared to 0.7 different prescriptions for the controls; this difference was significant (Table 2). Of the asthmatic children, 47% did not receive any asthma-related prescription at all in 2014. A high OR of 56.2 was observed for anti-asthmatics (R03). Examining R03 at the ATC 3 level, adrenergic inhalants (e.g. selective beta-2 adrenoreceptor agonists) were given to 46.1% of the children diagnosed with asthma during our 1-year observation period. Of the asthmatic children, 28.9% received (also) different inhalants (e.g. inhaled corticosteroids) for obstructive airway diseases. Only 2.0% of the children received other systemic drugs for airway diseases (e.g. leukotriene receptor antagonists). More than 3% received at least one short course of steroid tablets during the 1-year observation period (OR 12.0) (Table 4).

**Table 4:** Significantly (p≤0.001) associated medication in children registered with only asthma (As) versus controls (non-atopic children) (n = 15,774).

ICPC	OR	95%-CI	Prevalence (%)		OR per sex group		OR within age			Description ICPC codes
			As	No atopy	boy	girl	0–6	7–12	13–18	
**Atopic eczema related medication**										
D02	2.54	2.04–3.14	3.73	1.51						Emollients and protectives
D07	2.32	2.00–2.68	7.67	3.46						Corticosteroids, dermatological preparations
**Asthma related medication**										
H02	11.96	7.65–18.70	3.09	0.27						Corticosteroids for systemic use
R03	56.17	47.58–66.32	52.63	1.94			26.85	62.93	116.91	Anti-asthmatics ^†^
**Allergic rhinitis related medication**										
R01	4.61	3.99–5.34	12.49	3.00			2.65	5.41	5.47	Nasal preparations ^†^
R06	4.45	3.85–5.14	12.24	3.04			2.55	5.77	4.88	Antihistamines for systemic use ^†^
**Medication related to atopic disorders**										
P02	***1*.*36***	***0*.*81–2*.*29***	***0*.*43***	***0*.*32***	3.44	0.55				Anthelmintic *
D06	1.36	1.17–1.57	5.64	4.23						Antibiotics and chemotherapeutics for dermatological use
N06	1.41	1.21–1.65	5.07	3.66						Psychoanaleptic
M01	1.48	1.23–1.78	3.70	2.55						Anti-inflammatory and anti-rheumatic products
G03	1.49	1.25–1.77	6.25	5.01						Sex hormones and modulators of the genital system
A06	1.52	1.29–1.77	5.08	3.42						Laxatives
S02	1.59	1.32–1.90	3.84	2.46						Otologicals
S01	1.68	1.43–1.98	4.97	3.02						Ophthalmologicals
J01	1.81	1.65–1.98	18.21	11.03						Antibacterial for systemic use
N02	2.00	1.52–2.62	2.00	1.01						Analgesics
A02	2.03	1.49–2.76	1.55	0.77						Drugs for acid-related disorders
A03	2.28	1.61–3.23	1.32	0.58						Drugs for functional gastrointestinal disorders
R05	2.37	1.86–3.03	2.75	1.18						Cough and cold preparations
J07	5.69	4.32–7.49	4.23	0.77						Vaccines
C01	13.01	6.02–28.08	1.14	0.09						Cardiac therapy (e.g. epinephrine auto-injectors)

* significant (p≤0.01) influence of gender;

^†^ significant (p≤0.01) influence of age

**Italic**: overall model not significant

According to our analysis (Table 4), asthmatic children use more hormonal contraceptives (G03A) (5.9% vs. 4.6%), received more viral vaccines (4.2% vs. 0.8%) and used more ADHD-related medication (OR 1.4). These asthmatic children also received more analgesics prescribed by the GP (M01 and N02) compared to children without asthma. This will most likely concern the prescription of paracetamol and NSAIDs.

In asthmatic patients, anti-asthmatics (OR 26.9-> 116.9) were more often prescribed at older age.

Asthma-related medication was also prescribed for children that were not registered as having asthma. For anti-asthmatics about 0.8–6.2% of the children with atopic comorbidity received these prescriptions, compared to 0.3–2.1% of the non-atopic children. Medications related to atopic eczema (OR 2.3–2.5) and AR (OR 4.5–4.6) were more frequently prescribed for children with asthma (Table 4).

### Children registered with only allergic rhinitis

A child with AR received on average 2.2 different prescriptions in 2014, compared to 0.8 different prescriptions for the controls; this difference was significant (Table 2). Only 30% of these children did not receive any relevant AR prescription. High ORs are seen for medication prescribed by GPs to relieve AR symptoms (OR 21.4–40.8). Looking at the prescribed nasal preparations, these refer to R01A (OR 21.4; decongestants and other nasal preparations for topical use) and represent the prescription of anti-allergic agents and corticosteroids (Table 5).

**Table 5:** Significantly (p≤0.001) associated medication in children registered with only allergic rhinitis (AR) versus controls (non-atopic children) (n = 13,670).

ICPC	OR	95%-CI	Prevalence (%)		OR per sex group		OR within age			Description ICPC codes
			As	No atopy	boy	girl	0–6	7–12	13–18	
**Atopic eczema related medication**										
D02	3.36	2.66–4.25	4.46	1.38						Emollients and protectives
D07	2.74	2.34–3.22	8.38	3.23						Corticosteroids, dermatological preparations
**Asthma related medication**										
H02	3.26	1.89–5.62	0.80	0.25						Corticosteroids for systemic use
R03	4.42	3.55–5.51	6.20	1.48						Anti-asthmatics
**Allergic rhinitis related medication**										
R01	21.36	18.55–24.60	42.09	3.29						Nasal preparations
R06	40.77	35.02–47.46	53.59	2.78						Antihistamines for systemic use
**Medication related to atopic disorders**										
N03	***0*.*82***	***0*.*47–1*.*43***	***0*.*34***	***0*.*41***			0.50	0.20	1.25	Antiepileptic ^†^
D11	***1*.*27***	***0*.*91–1*.*76***	***1*.*19***	***0*.*94***			2.01	2.40	0.96	Other dermatological preparations ^†^
D06	1.38	1.16–1.65	4.49	3.29						Antibiotics and chemotherapeutics for dermatological use
J01	1.41	1.27–1.57	13.30	9.85						Antibacterial for systemic use
M01	1.43	1.22–1.67	5.98	4.30						Anti-inflammatory and anti-rheumatic products
D01	1.46	1.23–1.75	4.54	3.15						Antifungals for dermatological use
N02	1.51	1.18–1.92	2.44	1.64						Analgesics
A06	1.51	1.27–1.81	4.62	3.12						Laxatives
A02	1.68	1.28–2.19	2.11	1.27						Drugs for acid-related disorders
R05	1.80	1.40–2.31	2.55	1.43						Cough and cold preparations
S01	8.89	7.61–10.37	20.51	2.82						Ophthalmologicals
C01	10.31	3.69–28.80	0.60	0.06						Cardiac therapy (e.g. epinephrine auto-injectors)
V01	#	# – #	1,43	0,00						Allergens (e.g. immunotherapy)

^†^ significant (p≤0.01) influence of age;

^#^ OR could not be calculated

**Italic**: overall model not significant

Ophthalmological medications prescribed for these children refer to the prescription of anti-infectives (2.6% vs. 1.6%) and of anti-allergics (17.8% vs. 0.7%). Also, these children used more analgesics (M01 and N02) and systemic antibiotics (13.3% vs. 9.9%) compared to children without AR.

Sex or age did not influence the prescription of AR-related medication in children clearly with AR.

AR-related medication was also prescribed for children that were not registered as having AR. For AR-related medication about 4.7–12.5% of the children with atopic comorbidity received these prescriptions, compared to 2.8–3.1% of the non-atopic children. Medication related to atopic eczema (OR: 2.7–3.4) and asthma (OR: 3.3–4.4) were prescribed more frequently in children with AR (Table 5).

### Atopic triad

In total 559 children, who had all three atopic disorders, received more atopic-related prescriptions compared to non-atopic children (94% vs. 10%) (Table 6). Dermatological corticosteroids were prescribed more often for these children compared to non-atopic children (56.4% vs. 3.2%; OR 39.3). Also, the prescription of anti-asthmatics is much higher in these children compared to non-atopic children (68.3% vs. 1.3%; OR 176.1). This pattern is also seen for antihistamines (62.8% vs. 2.2%; OR 82.5). Antibiotics, especially penicillin and macrolides, were prescribed more frequently in children with all three atopic disorders.

**Table 6:** Significantly (p≤0.001) associated medication in children diagnosed with atopic triad (AT) (p≤0.001) (n = 1,118).

ATC	OR	95% CI	Prevalence (%)		Description ATC codes
			AT	No atopy	
**Atopic eczema related medication**					
D02	21.73	12.42–38.01	35.42	2.50	Emollients and protectives
D07	39.29	23.84–64.75	56.35	3.22	Corticosteroids, dermatological preparations
**Asthma related medication**					
H02	28.56	3.86–211.08	4.83	0.18	Corticosteroids for systemic use
R03	176.13	81.65–379.94	68.34	1.25	Anti-asthmatics
**Allergic rhinitis related medication**					
R01	36.84	20.74–65.45	46.69	2.33	Nasal preparations
R06	82.50	45.16–150.70	62.79	2.15	Antihistamines for systemic use
**Medication related to atopic disorders**					
J01	2.10	1.47–2.99	18.25	9.66	Antibacterials for systemic use
D06	2.93	1.71–5.02	9.30	3.40	Antibiotics and chemotherapeutics for dermatological use
S01	6.17	3.94–9.66	22.36	4.47	Ophthalmologicals
D11	6.28	2.16–18.24	4.29	0.72	Other dermatological preparations
J07	17.21	4.10–72.30	5.72	0.36	Vaccines
C01	#	# – #	5.90	0.00	Cardiac therapy (e.g. epinephrine auto-injectors)

^#^ OR could not be calculated

## Discussion

The present study shows that atopic children received both more atopic and non-atopic prescriptions, compared to non-atopic children. Age and gender did not clearly explain these differences. The prescriptions provided by a GP to relieve atopic symptoms seem to reflect preferred medication in relevant evidence-based medicine guidelines.

For atopic eczema the combination of emollients (cornerstone of the treatment) and corticosteroid crèmes are advised [3]. However, a corticosteroid crème was prescribed more frequently than an emollient. An explanation could be the freely available emollients at pharmacies or drugstores, which were not systematically registered in our database.

Anti-asthmatics are prescribed in accordance with the guidelines [4]. This clear reflection of the guideline could be the result of the policy that anti-asthmatics are not freely available. However, since inhaled corticosteroids are the cornerstone of asthma treatment, the relatively low use (29%) of inhaled corticosteroids (ICS) is remarkable. There are three possible explanations for this observation. Primarily, GPs will treat more children with mild intermittent asthma and don’t see more severe cases that justify (continuous) ICS use. Unfortunately, results from e.g. the ‘Asthma Control Questionnaire’ were not available to check this assumption. Secondly, there could be an overestimation of asthma diagnoses in the EHRs, since a proportion of the children will outgrow asthma. Finally, it could also reflect insufficient treatment, which could be supported by the observation that 3.1% received a short course of steroid tablets. All three explanations raise the question as to whether GPs adequately monitor the asthmatic children registered in their practice

Although oral antihistamines for AR are freely available, >70% of the patients still consult their GP for advice regarding AR-relevant medication. A systematic review [1] reported that the prevalence of AR in the open population, compared to the prevalence of AR in a primary care clinic, is much higher; therefore, we assume that only more severe cases visited the GP. This could explain the high number of prescriptions. Possibly because the free available antihistamines were not sufficient in the treatment of AR symptoms. Although the prescribed medication for AR also reflects the guideline [5], more information on the severity and type of symptoms of patients is needed to make a clearer judgement.

Finally, the existence of a fourth distinct group of atopic children is supported by the observation that children with all three atopic disorders receive more atopic-related prescriptions (94%) (with a distinct pharmacological profile) from their GP compared to non-atopic children or children with only one atopic disorder. This suggests that children with all three atopic disorders have a different phenotype. The GP seems to be aware of this, considering the high rate of prescriptions given to these children. However, since there is evidence for insufficient labeling of atopic disorders, this group might be even larger than observed in the present study.

This study shows that specific ATC codes are often prescribed for specific atopic disorders. Nevertheless, GPs did prescribe atopic-related medication to atopic children, even when they were not registered with that specific atopic disorder. Taking into account that the three atopic disorders are closely related, we postulate that when a child is already diagnosed with at least one atopic disorder and that child uses atopic-related medication for the other atopic disorders, it is plausible that the child will in fact have these other atopic disorders. For example, a child is diagnosed with eczema and receives anti-asthmatics, it is likely that this child will also have asthma. Non-atopic children also receive prescriptions for specific atopic-related medication. Both of these observations might reflect underdiagnosis, or at least insufficient registration. A different study design is needed to prove this hypothesis. According to Mulder et al. [10], children diagnosed with asthma can be reliably identified with a range of medication proxies. However, the use of prescription data for the identification of children diagnosed with atopic dermatitis and AR remains questionable.

This study also shows that atopic children received more non-atopic related medication. For example, the prescription of dermatologicals is particularly increased in children with atopic eczema. The main indication seems to be the treatment of skin infections (antifungals, antibiotics, antiseptics). In children with atopic eczema the skin barrier function is negatively affected, causing an increased risk of secondary skin infections. All atopic children received more oral antibiotic prescriptions. GPs either consider these children to be at increased risk for a complicated course of an infection, or these children indeed have more bacterial superinfections that justify the oral antibiotics. Antibiotics are particularly interesting to study, since their use is associated with an increased risk for the development of atopic disorders, in particular asthma [11, 12]. Or the relation between atopic disorders and antibiotics is a result of the confounding effect of early respiratory infections [13]. Future research should focus on the reason why these atopic children receive more antibiotics and whether this is indeed necessary. When examining the data in more detail, one specific pattern stands out. Although in absolute terms not frequently prescribed, there appears to be a stronger indication for the prescription of epinephrine auto-injectors (C01) in children with atopic disorders. The only indication for such medication is the treatment of anaphylaxis. Apparently, these children are at higher risk for the development of severe allergic reactions (possibly due to a food allergy or insect bites), a well-known comorbidity for atopic children. These IgE-mediated food allergies could also explain gastro-intestinal symptoms that are frequently observed in atopic children [14, 15], which might explain prescriptions related to the gastro-intestinal system (e.g. laxatives). The possibility that gastrointestinal symptoms might be a manifestation of adverse reactions to drugs prescribed for e.g. asthma and AR, was considered. However, Powel et al. [14] found this unlikely, as the prevalence of gastrointestinal symptoms in patients with asthma treated with inhaled adrenergics, inhaled corticosteroids or neither of these drugs, showed no significant differences. Unfortunately, the ICPC-1 coding system does not allow the registration of food allergies, so this could not be explored. Overall, atopic children receive more (different) prescriptions compared to non-atopic children, indicating that children with atopic disorders should be better monitored by their GP.

For the present study we used an extensive and representative general practice database [7]. The large numbers of atopic children that could be identified and matched with a non-atopic child gives the study substantial power and generalizability. This allowed evaluation of links between atopic disorders and rare prescriptions, such as ‘epinephrine auto-injectors’ and immunotherapy, both of which were associated with atopic disorders in this study. Using only data from general practices with sufficient data quality increases the reliability of this study. Furthermore, ATC codes were automatically attached when a GP prescribed medication using the electronical medical record system.

A limitation of the present study is related to which ICPC code the GPs uses for the episodes of the atopic disorders. For example, a child with a wheeze could be labeled either as ‘asthma’ (R96) or labeled as ‘wheeze’ (R03). This could result in both overestimation or underestimation of asthma. To decrease this risk of overestimation of atopic disorders, some episodes were corrected to select more severe cases. Furthermore, due to the hierarchical structure of the data (patients registered in general practices), a multi-level logistic regression analysis was performed to test whether clustering effects influenced our findings. Since this was not the case, only the results of the logistic regression analyses were presented. Another limitation regarding this type of explorative study is the unavoidable multiple testing. Therefore, a low p-value was used. Furthermore, the aim of this study was only to explore associations and interactions in atopic children and not to test specific hypotheses. Therefore, type 1 errors cannot be avoided; some associations emerging from this study might in fact reflect these type 1 errors such as antiepileptic and anthelmintic prescriptions. Finally, atopic children might visit the GP more frequently than non-atopic children. This can result in more prescriptions for atopic children and might partly explain some of the associations found. In future research, the number of prescriptions might need to be taken into account in the analyses.

In conclusion, the prescriptions provided by a GP to relieve atopic symptoms seem to reflect preferred medication in relevant evidence-based medicine guidelines. The present study shows that specific atopic-related prescriptions are prescribed for atopic as well as for non-atopic children that are not registered as having that specific atopic disorder. This observation might reflect underdiagnosis or at least insufficient registration and the GP needs to be aware of this. Overall, atopic children receive more (different) prescriptions compared to non-atopic children. This indicates that children with atopic disorders need better monitoring by their GP.

## Supporting information

S1 Table Studied ATC codes. (DOCX)
