# Supplementary material for: Atopic children and use of prescribed medication: A comprehensive study in general practice
Source: PLoS One. 2017 Aug 24;12(8):e0182664. doi: 10.1371/journal.pone.0182664 (PMC5570284; doi:10.1371/journal.pone.0182664)
Supplement: S1 Table — (DOCX) [file pone.0182664.s001.docx]

**S1 Table**

|  | |
| --- | --- |
|  | |
| **ATC Codes** | **Description** |
| **Alimentary tract and metabolism** |  |
| A01 | Stomatological preparations |
| A02 | Drugs for acid related disorders |
| A03 | Drugs for functional gastrointestinal disorders |
| A04 | Antiemetic and antinauseants |
| A05 | Bile and liver therapy |
| A06 | Laxatives |
| A07 | Antidiarrheal, intestinal anti-inflammatory/anti-infective agents |
| A08 | Antiobesity preparations, excluding diet products |
| A09 | Digestives, including enzymes |
| A10 | Drugs used in diabetes |
| A11 | Vitamins |
| A12 | Mineral supplements |
| A13 | Tonics |
| A14 | Anabolic agents for systemic use |
| A15 | Appetite stimulants |
| A16 | Other alimentary tract and metabolism products |
| **Blood and blood forming organs** | |
| B01 | Antithrombotic agents |
| B02 | Antihemorrhagics |
| B03 | Antianemic preparations |
| B05 | Plasma substitutes and perfusion solutions |
| B06 | Other haematological agents |
| **Cardiovascular system** | |
| C01 | Cardiac therapy |
| C02 | Antihypertensives |
| C03 | Diuretics |
| C04 | Peripheral vasodilators |
| C05 | Vasoprotectives |
| C07 | Beta blocking agents |
| C08 | Calcium channel blockers |
| C09 | Agents acting on the renin-angiotensin system |
| C10 | Lipid modifying agents |
| **Dermatologicals** | |
| D01 | Antifungals for dermatological use |
| D02 | Emollients and protectives |
| D03 | Preparations for treatment of wounds & ulcers |
| D04 | Antipruritics, incl antihistamines, anaesthetics, etc. |
| D05 | Antipsoriatics |
| D06 | Antibiotics and chemotherapeutics for dermatological use |
| D07 | Corticosteroids, dermatological preparations |
| D08 | Antiseptics and disinfectants |
| D09 | Medicated dressings |
| D10 | Anti-acne preparations |
| D11 | Other dermatological preparations |
| **Genito-urinary system and sex hormones** | |
| G01 | Gynaecological anti-infectives and antiseptics |
| G02 | Other gynaecologicals |
| G03 | Sex hormones and modulators of the genital system |
| G04 | Urologicals |
| **Systemic hormonal preparations, excluding sex hormones and insulins** | |
| H01 | Pituitary and hypothalamic hormones |
| H02 | Corticosteroids for systemic use |
| H03 | Thyroid therapy |
| H04 | Pancreatic hormones |
| H05 | Calcium homeostasis |
| **Anti-infective for systemic use** | |
| J01 | Antibacterials for systemic use |
| J02 | Antimycotics for systemic use |
| J04 | Antimycobacterials |
| J05 | Antivirals for systemic use |
| J06 | Immune sera and immunoglobulins |
| J07 | Vaccines |
| **Antineoplastic and immunomodulating agents** | |
| L01 | Cytostatics |
| L02 | Endocrine therapy |
| L03 | Immunomodulating agents |
| L04 | Immunosuppressive agents |
| **Musculo-skeletal system** | |
| M01 | Anti-inflammatory and anti-rheumatic products |
| M02 | Topical products for joint and muscular pain |
| M03 | Muscle relaxants |
| M04 | Antigout preparations |
| M05 | Drugs for treatment of bone diseases |
| M09 | Other drugs for disorders of the musculo-skeletal system |
| **Nervous system** | |
| N01 | Anaesthetics |
| N02 | Analgesics |
| N03 | Antiepileptics |
| N04 | Anti-Parkinson drugs |
| N05 | Psycholeptics |
| N06 | Psychoanaleptics |
| N07 | Other nervous system drugs |
| **Antiparasitic products, insecticides and repellents** | |
| P01 | Antiprotozoals |
| P02 | Antihelmintics |
| P03 | Extoparasiticides, incl. scabicides, insecticides and repellents |
| **Respiratory system** | |
| R01 | Nasal preparations |
| R02 | Throat preparations |
| R03 | Anti-asthmatics |
| R05 | Cough and cold preparations |
| R06 | Antihistamines for systemic use |
| R07 | Other respiratory system products |
| **Sensory organs** | |
| S01 | Ophthalmologicals |
| S02 | Otologicals |
| S03 | Ophthalmologicals and otologicals preparations |
| **Various** |  |
| V01 | Allergens |
| V03 | All other therapeutic products |
| V04 | Diagnostic agents |
| V06 | General nutrients |
| V07 | All other non-therapeutic products |
| V08 | Contrast media |
| V09 | Diagnostic radiopharmaceuticals |
| V10 | Therapeutic radiopharmaceuticals |
